# Acute toxicity of definitive chemoradiation in patients with inoperable or irresectable esophageal carcinoma

**DOI:** 10.1186/1471-2407-14-56

**Published:** 2014-01-31

**Authors:** Nadia Haj Mohammad, Maarten CCM Hulshof, Jacques JGHM Bergman, Debby Geijsen, Johanna W Wilmink, Mark I van Berge Henegouwen, Hanneke WM van Laarhoven

**Affiliations:** 1 Department of Medical Oncology, Academic Medical Center, University of Amsterdam, Amsterdam, the Netherlands; 2 Department of Radiation Oncology, Academic Medical Center, University of Amsterdam, Amsterdam, the Netherlands; 3 Department of Gastroenterology and Hepatology, Academic Medical Center, University of Amsterdam, Amsterdam, the Netherlands; 4 Department of Surgery, Academic Medical Center, University of Amsterdam, Amsterdam, the Netherlands

**Keywords:** Definitive chemoradiation, Esophageal cancer, Inoperable, Irresectable, Toxicity

## Abstract

**Background:**

Definitive chemoradiation (dCRT) is considered curative intent treatment for patients with inoperable or irresectable esophageal cancer. Acute toxicity data focussing on dCRT are lacking.

**Methods:**

A retrospective analysis of patients treated with dCRT consisting of 6 cycles of paclitaxel 50 mg/m2 and carboplatin AUC2 concomitant with radiotherapy (50.4 Gy\1.8Gy) from 2006 through 2011 at a single tertiary center was performed. Toxicity, hospital admissions and survival were analysed.

**Results:**

127 patients were treated with definitive chemoradiation. 33 patients were medically inoperable, 94 patients were irresectable, Despite of a significantly smaller tumor length in inoperable patients grade ≥3 toxicity was significantly recorded more often in the inoperable patients (44%) than in irresectable patients (20%) (p < 0.05) Hospital admission occurred more often in the inoperable patients (39%) than in the irresectable patients (22%) (p < 0.05) Median number of cycles of chemotherapy was five for inoperable patients (p = 0.01), while six cycles could be administered to patients with irresectable disease. Recurrence and survival were not significantly different. The odds ratio for developing toxicity ≥ grade 3 was 2.6 (95% CI 1.0-6.4 p < 0.05) for being an inoperable patient and 1.2 (95% CI 1.0-1.4 p = 0.02) per 10 extra micromol/l creatinine.

**Conclusions:**

Our data show that acute toxicity of definitive chemoradiation is worse in patients with medically inoperable esophageal carcinoma compared to patients with irresectable esophageal cancer and mainly occurs in the 5th cycle of treatment. Improvement of supportive care should be undertaken in this more fragile group.

## Background

Esophageal carcinoma is the eighth most common cancer worldwide [1]. The total incidence of esophageal cancer is rising, mainly as the result of a marked increase in the incidence of adenocarcinoma. It is often diagnosed in late stages, and approximately 50% of the patients have potentially curable disease.

In patients who are considered fit for surgery and have technically resectable disease the treatment of choice is surgical resection. Outcome of esophageal resection can be improved by multimodality treatment. A meta-analysis has shown significant benefit of chemoradiation followed by over surgery alone for both adenocarcinoma and squamous cell carcinoma. The hazard ratio for all-cause mortality with neoadjuvant chemoradiotherapy versus surgery alone was 0.81 (95% CI 0.70-0.93; p = 0.002), corresponding to a 13% absolute difference in survival at 2 years [2]. In the Netherlands the preferred radiochemotherapy regimen consists of carboplatin plus paclitaxel concurrent with 41.4 Gy of radiation, which is based on the results of the Dutch CROSS study which showed a median survival of 49.4 months in the chemoradiotherapy surgery arm versus 24 months in the surgery group. The chemoradiation regimen was well tolerated with 7% hematologic and 13% non hematologic grade 3/4 toxicities, mainly leukopenia and anorexia [3].

In contrast to patients with resectable disease, patients staged irresectable (T4N0-1 M0) by endoscopic ultrasonography (EUS) have a very poor prognosis [4,5]. Primary surgery does not prolong survival and patients can be treated with chemotherapy, radiation therapy or best supportive care [6,7].

Based on the results of the RTOG 8501 [8] chemoradiotherapy is superior compared to radiotherapy alone. In this prospective randomized clinical trial, patients with squamous cell carcinoma or adenocarcinoma were treated with chemoradiotherapy (4 cycles of 5-fluorouracil and cisplatin and radiotherapy (50 Gy at 2 Gy/d) or radiotherapy alone (64 Gy). The combined modality arm demonstrated a significant improvement in both median survival (14 vs. 9 months) and 5-year overall survival (27% vs. none) with projected 8-year and 10-year survival rates of 22% and 20% respectively.

Only a few studies described the toxicity results of the definitive chemoradiation for irresectable tumors (T4N0-1 M0). In a German study 22 patients were treated with induction chemotherapy with 5FU and cisplatin followed by concurrent chemoradiation therapy for T4 and obstructing T3 squamous cell carcinoma of the upper and midthoracic esophagus. A partial or complete response was seen in 9 (41%) and 1 (5%) patients, respectively, and 41% of the patients were alive at 2 years after treatment. The main toxicities in this cisplatin based chemoradiation scheme were leukocytopenias (23%) as well as thrombocytopenias (9%) grade III and IV. A total of 10 patients (45%) had grade III and IV dysphagia during chemoradiation (in 32% of the patients this was already preexistent) [9].

Given the favourable toxicity profile of chemoradiation with carboplatin and paclitaxel in the preoperative setting, a phase II study was performed in the Netherlands with concurrent chemoradiation with paclitaxel and carboplatin as definitive treatment for patients with irresectable esophageal cancer. Durable locoregional control and palliation was achieved in about half of the patients. Median overall and disease-free survival were 17 months and 9 months respectively [10]. The main grade 3–4 toxicities were neutropenia (16%) thrombocytopenia (4%), esophagitis (12%) and (fatigue (8%).

Since 2003 patients with irresectable esophageal cancer as well as patients medically unfit for surgery, but deemed fit for definitive chemoradiation were treated in our centre with this treatment scheme. The tolerability of this chemoradiation treatment regimen for medically inoperable patients has never been described in a substantial cohort of patients.

Therefore, in this retrospective cohort study we compare acute toxicity of the definitive chemoradiation with carboplatin and paclitaxel in patients with medically inoperable esophageal cancer and patients with irresectable esophageal cancer.

## Methods

### Patients and study design

The medical charts of all patients with esophageal and esophagastric junction cancer treated between March 2006 and October 2011 in the Academic Medical Centre with definitive chemoradiation were retrieved. Treatment strategy was defined during multidisciplinary meetings for all patients. Patients were treated with carboplatin targeted at an area under the curve (AUC) of 2 mg per milliliter per minute and paclitaxel 50 mg per square meter of body surface area. Carboplatin and paclitaxel were administered intravenously on days 1, 8, 15, 22, 29, and 36. A total radiation dose of 50.4 Gy was given in 28 fractions of 1.8 Gy, in 5 fractions administered per week. All patients were treated by means of external beam radiation.

Patients were divided into two groups based on the reason for treatment with definitive chemoradiation: inoperable patients and irresectable patients. Inoperable patients were defined as patients with surgical contraindications like heart failure NYHA III and IV, FEV1 <1.5 L or WHO performancescore 3 or 4. Irresectable patients were defined as patients with locally irresectable carcinoma of the esophagus or gastric junction (T4N0-1 M0) [11], patients with locally recurrent carcinoma of the esophagus outside the prior radiation field, a cervical carcinoma of the esophagus or patients with involvement of celiac or supraclavicular lymphnodes (M1a). When more than one reason was given for inoperability the predominant factor is stated. Patients refusing surgery and treated with dCRT instead were excluded from analyses.

### Data collection

According to national regulations formal ethics approval for researching human data according was not needed [12,13]. We recorded clinical, histopathological, laboratory and endoscopic data from the hospital records.

At baseline, age, tumor length, comorbidity, body surface area (BSA), Body Mass Index (BMI) and WHO performance score were noted. Furthermore, tumor histology (squamous carcinoma, adenocarcinoma and other) as well as baseline creatinine (micromol/liter) and albumin (U/l) were scored.

Toxicity throughout chemoradiation was scored according to Common Terminology Criteria for Adverse Events version 3.0. Also dose modifications or discontinuation of chemo- and radiotherapy were recorded.

Locoregional recurrences and distant metastasis were defined by proven histology of the recurrent site, or by clinical signs of recurrent disease combined with progression on CT scan or PET/CT scan or suspicious endoscopic findings.

### Statistical analyses

Descriptive statistics of categorical variables are reported as total numbers and percentages. Continuous variables are reported as means and standard deviation. To assess whether irresectable and inoperable patients differed in baseline characteristics unpaired t tests were used for continuous variables with normal distribution. For variables with non-normal distribution the Mann Whitney test was performed. To compare toxicity between the two groups the chi square test was performed.

To identify other determinants for toxicity than having inoperable or irresectable esophageal cancer we performed logistic regression analysis. We selected those determinants that either differed significantly between the inoperable and inresectable groups at baseline, and/or showed a significant univariable relation with toxicity. These determinants were used in separate multivariable models, each model including one determinant and the main group factor (inoperabel versus inresectable).

The Kaplan-Meier method was used to estimate survival, with the log rank test to determine significance.

All statistical analyses were performed with SPSS (version 19.0). Statistical inferences were based on 2-sided tests with p < 0.05 considered to be statistically significant.

## Results

### Patient characteristics

Overall, 127 patients were treated with definitive chemoradiation in our institution. Distribution of patients in the inoperable and irresectable group are listed in Table 1. Characteristics of these patients are listed in Table 2.

**Table 1:** Distribution of patients in inoperable and irresectable group

**Reason inoperable n = 33**	
Comorbidity cardiovascular n (%)	2 (6%)
Comorbidity respiratory n (%)	14 (42%)
Comorbidity performancestatus n (%)	10 (30%)
Comorbidity other n (%)	7 (2%)
**Reason irresectable n = 94**	
T4a n (%)	15 (16%)
T4b n (%)	9 (10%)
Recurrent carcinoma n (%)	19 (20%
Cervical carcinoma n (%)	9 (10%)
M1a disease n (%)	41 (44%)

**Table 2:** Baseline characteristics of inoperable and irresectable patients

	**Inoperable group**	**Irresectable group**	**P value**
N = 127	33	94	
Sex			0.21
Male n (%)	20 (61%)	68 (72%)	
Female n (%)	13 (39%)	26 (28%)	
Age (years) (mean and SD)	72 (9)	63 (10)	0.00
Range	53-89	25-84	
BMI (kg/m2) (mean and SD)	24.1 (5.4)	23.6 (3.6)	0.65
BSA (m2) (mean and SD)	2.0 (0.6)	1.8 (0.2)	0.16
Tumor length (cm) (mean and SD)	5.2 (2.7)	6.5 (2.7)	0.04
Stage I/II	13 (40.6%)	n.a.	
Stage III/IV	19 (59.4%)	100%	
Missing	1		
Histology			0.14
Adenocarcinoma	18 (55%)	35 (37%)	
Squamous cell car.	12 (36%)	53 (56%)	
NNO	3 (9%)	6 (6%)	
Laboratory			
Creatinine μmol/l (median and IQR)	79 (64–105)	70 (60–82)	0.03
Albumin U/l (mean and SD)	43 (4)	43 (4)	0.99
Hemoglobin mmol/l (mean and SD)	8.6 (1.0)	8.4 (1.0)	0.38

SD: standard deviation.

IQR interquaertile range.

n.a. not applicable.

The mean age of patients at diagnosis was 72 years in the inoperable group and 63 years in the irresectable group (p < 0.01) Males were predominant in both groups.

Inoperable patients had a significant higher creatinine compared to irresectable patients (86 micromol/l and 73 micromol/l, respectively; p < 0.05).

The average length of the primary tumor in the inoperable group was significantly shorter with a mean tumor length of 5.1 cm (SD 2.6 cm) (p <0.05). The irresectable group had a mean tumor length of 6.6 cm (SD 2.7 cm).

### Toxicity

Overall grade ≥3 toxicity was observed in 27% of the patients. Grade ≥3 toxicity occurred significantly more often in inoperable patients (44%) versus irresectable patients (20%, p <0.01). The main grade ≥ 3 toxicities were: leukopenia, neutropenia, esophagitis and fatigue (Table 3).

**Table 3:** Grade ≥ 3 toxicity in inoperable and irresectable patients

	**Inoperable group**	**Irresectable group**
Number of patients (%)	n = 33	n = 94
Anorexia	0 (0)	1 (1)
Diarrhea	1 (3)	0 (0)
Fatigue	2 (6)	3 (3)
Leukopenia	6 (18)	3 (3)
Neutropenia	3 (9)	0 (0)
Oesophagitis	5 (15)	9 (10)
Esophageal perforation	0 (0)	1 (1)

Six patients died during chemoradiation, one because of neutropenic sepsis, and five because of cardiac failure (two patients from the inoperable group and four patients from the irresectable group).

Overall 26% of patients were admitted non-electively to the hospital because of treatment toxicity. Non-elective hospital admission was observed significantly more often in the inoperable group (39% of patients) compared to the irresectable group (22%, p < 0.05). The most important reasons for hospital admission were dehydration and neutropenic fever.

In both groups full radiation doses could be administered to the majority of patients (85% of inoperable patients, 93% of irresectable patients, p = 0.19). However, 51% of patients with inoperable esophageal cancer and 27% of irresectable patients did not complete six cycles of chemotherapy (p = 0.01). Median number of cycles of chemotherapy was five for inoperable patients while on average six cycles could be administered to patients with irresectable disease (p = 0.01) (Table 4).

**Table 4:** Toxicity and completion of treatment in inoperable and irresectable patients

	**Inoperable group**	**Irresectable group**	**P value**
Number of patients	33	94	
Toxicity grade ≥ 3	14 (44%)	17 (20%)	0.01
WHO performance score ≥ 3	14 (45%)	21 (25%)	0.04
Hospital admittance (%)	13 (39%)	19 (22%)	0.048
Hospital stay (days) (IQR)	0 (0–5)	0 (0)	0.11
Chemotherapy complete (%)	16 (49%)	64 (73%)	0.01
Radiotherapy complete (%)	28 (85%)	87 (93%)	0.19
Median cycles (IQR)	5 (4–6)	6 (5.5-6.0)	0.00

To assess the influence of other determinants for toxicity we performed logistic regression analysis. Due to the relative small number of events (presence of grade ≥ 3 toxicity is 20-44%), extensive multivariable analysis was not possible. Age, creatinine, tumor length and T4 stadium (yes or no) differed significantly between the two groups (Table 2). Results of the univariate analysis between the baseline determinants and toxicity are shown in Table 5. Univariable prognostic factors for grade ≥ 3 toxicity were being an inoperable patient, age and creatinine level.

**Table 5:** Univariate analysis, determinants for developing toxicity grade ≥3

**Variable**	**Odds ratio**	**p value**	**95% confidence interval**
Inoperable patient	3,115	0,011	1,294-7,463
Age	1,049	0.036	1,003-1,098
Kreatinine	1,023	0.007	1,006-1,040
BMI	0,986	0.783	0,891-1,091
Tumor length	0,958	0,638	0,801-1,146
T4 stadium	1,169	0,755	0,438-3,122
Albumin	1,025	0,770	0,869-1,209

Table 6 shows the results of the multivariable analyses correcting for the effect of age, creatinine, tumor length and tumor stadium. Prognostic factors for grade ≥ 3 toxicity were being an inoperable patient (p = 0.045) and a high creatinine level (p = 0.021). The odds ratio for inoperable patients developing grade ≥ 3 toxicity was 2.68 (confidence interval 1.01-6.77.5). The increased odds for developing toxicity per 10 extra micromol/l creatinine was 1.23 (CI 1.01-1.45). After correction for the effect of age (Table 6) and tumor length (Table 6) the prognostic effect of inoperable patient on toxicity showed a trend for significance (p<. 10) and the odds ratio was high (>2.4) in all four corrected analyses.

**Table 6:** Multivariate odds ratio’s for determinants for developing grade ≥3 toxicity

	**Variable**	**Odds ratio**	**95% confidence interval**		**p value**
Model 1	Creatinine (per 10 micromol/l)	1.213	1.027	1.432	0.023
	Inoperable patient	2.551	1.021	6.369	0.045
Model 2	Age	1.034	0.985	1.084	0.174
	Inoperable patient	2.457	0.965	6.289	0.059
Model 3	Tumor length	0.986	0.822	1.183	0.877
	Inoperable patient	2.410	0.906	6.410	0.078
Model 4	Tumor stadium	1.560	0.501	4.856	0,443
	Inoperable patient	3.704	1.307	10.526	0.014

### Local recurrence and/or metastasis

Overall, local recurrence occurred in 42% of the patients and distant metastasis in 44% of the patients. No significant difference in local recurrence rate was observed between inoperable (27%) and irresectable patients (47%) (p = 0.12), nor for distant metastasis (p = 0.12; inoperable 31%, Irresectable 48%). 51% patients with local recurrence had synchronous distant metastasis.

### Survival

Median follow up was 44.5 months in the inoperable group and 51.9 months in the irresectable group. Survival in the inoperable and irresectable group was not significantly different (median overall survival 17.1 months versus 17.4 months, respectively (p = 0.89).

## Discussion

To our knowledge this is the largest retrospective study reporting on toxicity of definitive chemoradiation in patients with inoperable and irresectable esophageal cancer. We observed a significantly higher grade ≥ 3 toxicity in the patients that were medically inoperable compared to patients that were irresectable, resulting in significantly more hospital admissions and incomplete administration of chemotherapy.

Interestingly in our series the patients with inoperable esophageal carcinoma had a significantly shorter tumor length and consequently a smaller radiation field. Furthermore in the inoperable group 40% of the patients had low stage disease with stadium I-II disease. In despite of a smaller radiation field and lower tumor stage the observed toxicity was significantly higher.

In the literature a range of toxicity grade 3 or higher in definitive chemoradiation is observed from 11% to 50% of the patients depending on the proportion of patients with T4 disease [14-17]. Of note, the majority of these studies used the 5FU cisplatin scheme for which the main observed toxicities were neutropenia, mucositis, diarrhoea and neuropathy [3,10,14,15].

In order to complete the scheduled chemotherapy toxicity grade ≥ 3 should be minimized and hospital admission reduced. The most important reasons for the majority of hospital admissions were dehydration as a result of mucositis or esophagitis and neutropenic fever [16].

To prevent dehydration the start of enteric feeding in an early stage could be a feasible treatment option. Moreover, during chemotherapy administration, from cycle 5 onwards standard extra intravenous fluid suppletion could be considered. Finally, the introduction of prophylactic quinolones, starting from the fifth cycle, could be considered to prevent neutropenic fever in this patient population.

Failure after treatment of esophageal cancer in our series concerns both local recurrence and distant metastases in comparable frequencies without significant differences between inoperable patients and irresectable patients. The increased locoregional and distant recurrence rate (although not significant) in irresectable patients reflect the higher tumor stage in this group. Therefore, the lower toxicity in the irresectable patient cannot be explained by favourable tumor characteristics or smaller radiation field sizes. Half of the patients with distant failure have concurrent locoregional recurrence. This suggests that not only optimization in the administration of chemotherapy could be instrumental in the improvement of treatment outcome in patients with esophageal cancer receiving definitive chemoradiation, but also approaches aimed at reducing the risk of local relapse. For example, an esophagectomy after definitive chemoradiation or an integrated boost on the primary tumor could improve local control. These approaches are subject of ongoing Dutch studies. (NTR 3060, NTR3532).

Some limitations to our study should be noted. Data were retrospectively collected which may have resulted in an underestimation of toxicity occurrence. Furthermore, toxicity grade 1 and 2 were not scored in detail. The study reports on a single institute experience, which may not be representative of all practices that use the same chemoradiation scheme. Finally, the focus of this research was acute toxicity of definitive chemoradiation. Although survival between the two groups was not significantly different between the inoperable and irresectable group no definitive conclusions on survival can be drawn from this study.

## Conclusion

This is the first large study reporting on grade 3–4 toxicity of definitive chemoradiation with carboplatin and paclitaxel in patients with inoperable or irresectable esophageal cancer. Our data show that toxicity is worse in the inoperable group compared to the irresectable group and mainly occurs in the 5th cycle of treatment. Therefore, improvement of supportive care should be undertaken in this more fragile group to minimize side effects, preventing incomplete administration of chemotherapy, and thereby possibly prolonging survival.

## Competing interests

The authors declare that they have no competing financial or other interests.

## Authors’ contributions

NHM participated in the design, acquired the data, performed the statistical analysis and drafted the manuscript, HL performed the statistical analysis, monitored quality control of data and revised the manuscript, MH participated in the design, monitored quality control of data and revised the manuscript. JW participated in the design and revised the manuscript. JB, EG and MB revised the manuscript critically for important intellectual content. All authors read and approved the final manuscript.

## Pre-publication history

The pre-publication history for this paper can be accessed here:

http://www.biomedcentral.com/1471-2407/14/56/prepub
